# Vitreomacular Interface Abnormalities in Myopic Foveoschisis: Correlation With Morphological Features and Outcome of Vitrectomy

**DOI:** 10.3389/fmed.2021.796127

**Published:** 2022-01-05

**Authors:** Dong Fang, Li Wang, Lu Chen, Jia Liang, Kunke Li, Xingxing Mao, Ting Xie, Shaochong Zhang

**Affiliations:** ^1^Shenzhen Eye Hospital, Shenzhen Key Laboratory of Ophthalmology, Jinan University, Shenzhen, China; ^2^Department of Ophthalmology, Chengdu Second People's Hospital, Chengdu, China

**Keywords:** myopic foveoschisis, vitreomacular interface abnormalities, pars plana vitrectomy, morphological feature, optical coherence tomography

## Abstract

**Purpose:** To compare the morphologic characteristics and response to surgery of myopic foveoschisis (MF) with different patterns of vitreomacular interface abnormalities (VMIAs).

**Methods:** In this observational case series, 158 eyes of 121 MF patients with epiretinal membrane (ERM) or vitreomacular traction (VMT) based on optical coherence tomography (OCT) were enrolled. All the eyes were divided into two groups by the pattern of VMIAs: ERM and VMT group. Sixty-one eyes underwent pars plana vitrectomy (PPV) and were followed up for at least 6 months. The morphologic characteristics based on OCT and the surgical outcome were evaluated.

**Outcome:** ERM and VMT were found in 47.47 and 52.53% of the cases, respectively. A higher rate of foveal detachment (61.4 vs. 26.7%; *p* < 0.001) and a higher rate of outer lamellar macular hole (45.8 vs. 21.3%; *p* = 0.001) were detected in the eyes with VMT compared with those with ERM. In contrast, a lower rate of inner lamellar macular hole (28.9 vs. 60.0%; *p* = 0.001) was detected in the eyes with VMT compared with those with ERM. The disruption of the external limiting membrane (ELM) was more common in the eyes with VMT than in those with ERM (45.8 vs. 21.3%; *p* = 0.001). PPV was performed in 61 eyes with a mean follow-up time of 23.55 ± 19.92 months. After surgery, anatomical resolution was achieved in 51 eyes (83.6%). At the final visit, the mean central foveal thickness (CFT) decreased significantly from 547.83 to 118.74 μm, and the mean LogMAR BCVA improved significantly from 0.92 to 0.57. The VMT group was associated with a higher proportion of eyes with visual acuity improvement postoperatively (*p* = 0.02) and had more a decrease of CFT (*P* = 0.007) compared with the ERM group.

**Conclusion:** In the eyes with MF, outer retinal lesions occurred more frequently in the eyes with VMT, whereas inner retinal lesions occurred more frequently in the eyes with ERM. Tangential force generated by ERM may act as a causative factor for the inner retinal lesions in MF, and inward-directed force resulting from VMT may act as a causative factor for outer retinal lesions in MF.

## Introduction

Myopic foveoschisis (MF) is a common sight-threatening complication in patients with high myopia, which is characterized by intraretinal splitting in the macula region. It is reported that 9.3–31.3% of the highly myopic eyes with posterior staphyloma (PS) have foveal retinoschisis, which is believed to be one of the main causes of full-thickness macular holes (MHs) in highly myopic eyes, along with obvious vision impairment (1, 2).

Although the exact pathogenesis of MF has not been clarified, several factors have been found to play a role in the occurrence of MF, including vitreomacular interface abnormalities (VMIA), retinal arteriolar stiffness, globe ectasia and staphyloma. Among them, VMIAs including epiretinal membrane (ERM) and vitreomacular traction (VMT) are among the independent factors associated with MF (3). Different subtypes of VMIAs, with different tomographic presentations, could lead to different tractional forces on retina. In the eyes with VMT, contraction of the preretinal vitreous could lead to a trampoline-like shallow vitreous detachment, which generates anterior traction on the fovea (4). On the other hand, ERM, presented as a thin and highly reflective epiretinal material in optical coherence tomography (OCT), is proposed to generate tangential forces on the retinal surface (5).

Myopic foveoschisis may exhibit different anatomical configurations, including foveal detachment (FD), outer lamellar macular hole (OLMH), and inner lamellar macular hole (ILMH). It is reported that MF with FD presented poorer visual acuity and foveal sensitivity (6). Due to different directions of tractional forces, subtypes of VMIAs may have clinical implications on the anatomical configuration of MF. However, a clear distinction between the subtypes of VMIAs associated with the morphological and functional features of MF remains difficult.

Pars plana vitrectomy (PPV) has become the mainstay surgical treatment for MF by relieving the tractional forces generated by different VMIAs (7). However, the variation of surgical outcome in MF after PPV suggests that the subtypes of VMIAs might have an impact on the surgical response of MFs. Nevertheless, the relationship between the subtypes of VMIAs and the surgical outcome of MF is rarely described in the literature.

Therefore, in this study, we specifically enrolled MF patients with different subtypes of VMIAs, compared their morphologic characteristics and surgical outcome. By analyzing the anatomical configurations of MF with different subtypes of VMIAs, our goal was to elucidate the potential role of VMIAs in the pathogenesis of MF. The possible interrelationships between them were also analyzed from a mathematical viewpoint in this study.

## Methods

This study was approved by the Institutional Review Board of Shenzhen Eye Hospital (Shenzhen China) and was conducted in accordance with the World Medical Association Declaration of Helsinki. We retrospectively enrolled 158 eyes of 121 consecutive patients from January 2013 to January 2021. Inclusion criteria were: (1) Spherical equivalent (SE) ≤ −6 D or axial length (AL) ≥ 26.5 mm; (2) Diagnosed as foveoschisis by OCT; (3) Diagnosed as ERM or VMT by OCT. Exclusion criteria were: (1) Presence of full-thickness macular hole, (2) history of ocular trauma or vitreoretinal surgery, including vitrectomy and scleral reinforcement, (3) eyes with any associated or concomitant retinopathy that could confound the retinal interpretation of OCT images, (4) to dig out the relationship between the subtypes of VMIAs and MF, we specially enrolled the eyes with VMT alone or ERM alone, and the eyes with both VMIAs were excluded, (5) unclear image of OCT, which was defined as insufficient visualization of the retinal pigment epithelium line in the macular area.

### Ocular Examinations

**Figure 1:**
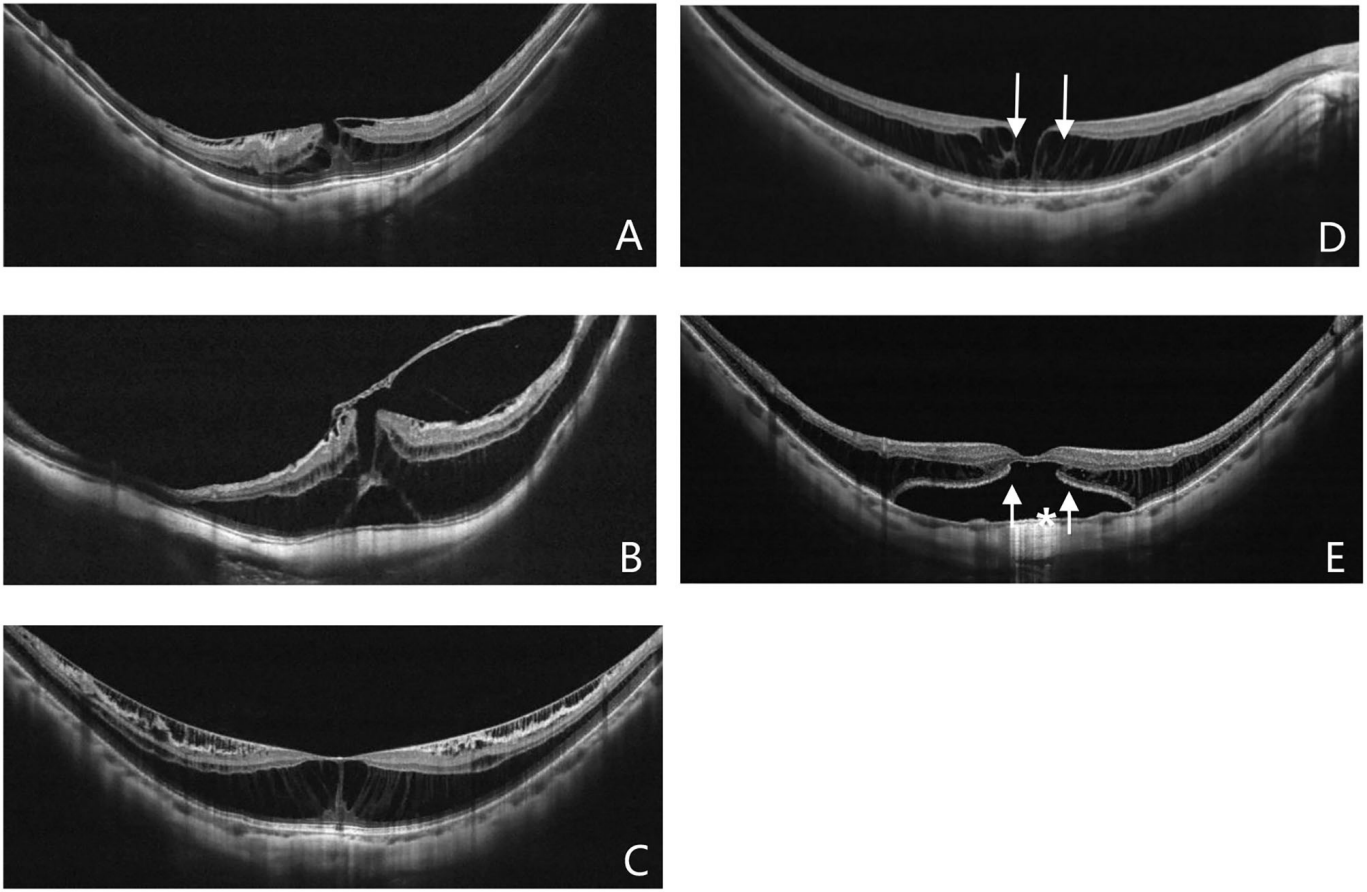
Different pattern of VMIAs **(A,B)**: Epiretinal membrane **(A)**, Vitreomacular traction **(B)**. The anatomical configurations of myopic foveoschisis (MF) in optical coherence tomography (OCT) **(C–E)**: MF alone **(C)**, inner lamellar macular hole (**D**, white arrow), outer lamellar macular hole (**E**, white arrow), foveal detachment (**E**, white asterisk).

All patients underwent comprehensive ophthalmologic examination, including best-corrected visual acuity (BCVA), slit-lamp microscope, dilated fundus examination, the measurement of refractive error by auto refractometer, AL by IOL master (Zeiss IOL Master; Carl Zeiss AG, Oberkochen, Germany), the presence of PS by Scanning Laser Ophthalmoscope (SLO), and central foveal thickness (CFT) by OCT (3D OCT-1000; Topcon Corporation, Tokyo, Japan or Spectralis OCT; Heidelberg Engineering GmbH, Heidelberg, Germany; Optovue, Inc., Fremont, CA, USA; because of the OCT equipment update in our hospital). All patients were divided into two groups by the pattern of VMIAs based on OCT examination: ERM group and VMT group. VMT is defined as the eyes with detectable retinal anatomic changes that occur on OCT and concurrent perifoveolar posterior vitreous detachment. Furthermore, VMT was divided into focal VMT (the area of attachment is 1,500 μm or less) or broad VMT (the area of attachment is more than 1,500 μm) (8). The BCVA, CFT, SE, AL, and the anatomical configurations of MF in OCT, including the presence of FD, OLMH, ILMH, the integrity of external limiting membrane (ELM), and the ellipsoid zone (EZ) were compared between the two groups (Figure 1). The EZ was classified as either intact or disrupted. The intact EZ had a continuous hyperreflective line over the retinal pigment epithelium. The disrupted EZ instead had a severely attenuated line. The status of ELM was interpreted in the same way. The outer retinoschisis was graded according to the location and the size, as suggested by Shimada et al. (9): no macular retinoschisis (S0); extrafoveal macular retinoschisis (S1); fovea-only macular retinoschisis (S2); foveal but not the entire macular area macular retinoschisis (S3); and entire macular area macular retinoschisis (S4). To control for subjective biases, the OCT examinations were independently interpreted by three different researchers. In case of disagreement in interpretation among the three researchers, the other two researchers reviewed the images in question. The results represent those with the highest unanimous approval from the five researchers.

### Surgical Subjects and Procedures

Among the whole group, 61 eyes of 56 patients were treated by PPV due to MF-caused vision decline or metamorphopsia, and they completed no <6 months of follow-up postoperatively. All the surgeries were done under retrobulbar anesthesia or general anesthesia by one experienced surgeon (S.Z.). Three-port 23-gage, 25-gauge, or 27-gauge pars plana vitrectomy was performed. After core vitrectomy, manual posterior vitreous detachment was induced. The ILM was stained by 0.1 ml of indocyanine green solution at a concentration of 0.5%. If ERM was present, it was removed first, then the ILM was peeled completely within the vascular arcades. Finally, fluid–air exchanges was performed, followed by 16% perfluoropropane (C3F8) or filtered air tamponade. After surgery, all the patients were required to keep a face-down position for at least 12 h/day for no <2 weeks in patients tamponaded with C3F8 or 1 week with filtered air. At postoperative follow-up, regular ophthalmic examination and OCT were performed. BCVA, CFT, the process of anatomical resolution, and operative complications were recorded.

### Statistical Analysis

Anatomical resolution was defined as complete foveal reattachment and the absence of foveoschisis. A postoperative gain or loss of at least two lines of Snellen visual acuity was considered statistical improvement or deterioration, otherwise defined as unchanged vision. Snellen visual acuity values were converted to the logarithm of the minimum angle of resolution (logMAR) for statistical analysis. Parametric data were presented as mean ± SD (standard deviation). SPSS 24.0 statistical software (SPSS Inc, Chicago, IL) was used for statistical analysis, with *p* < 0.05 considered statistically significant. The statistical comparison of the changes in visual acuity and CFT were analyzed with the independent sample *t*-tests or the Mann–Whitney test. The chi-square test or Fisher exact probability test was used to compare qualitative variables.

## Results

### Clinical Characteristics

A total of 213 eyes with MF were founded. Although 48 of the 213 eyes without ERM or VMT were excluded, seven eyes were excluded because of the poor quality of their OCT. Consequently, 158 eyes (121 patients) met the stated criteria and were included in the analysis. There were 51 men and 70 women. The mean age was 53.60 ± 10.63 (22–73) years, the mean refractive error was −13.19 ± 4.41 (−6.00 to −26.00) D, the mean axial length was 29.33 ± 1.59 (25.90–34.32) mm. Average logMAR BCVA was 0.85 ± 0.45 (0–1.85) and CFT was 507.60 ± 187.68 (105.00–1640.50) μm. PS existed in 145 eyes (91.8%), and dome-shaped macula (DSM) existed in three eyes (1.9%). All the eyes presented foveal retinoschisis. None of the eyes exhibited grade S1 outer retinoschisis, 28 eyes (17.7%) displayed retinoschisis limited to the foveal region (grade S2 outer retinoschisis), 50 eyes (31.6%) showed grade S3 outer retinoschisis, and 80 eyes (50.6%) had grade S4 outer retinoschisis.

### Vitreoretinal Interface Abnormalities

Epiretinal membrane and VMT were found in 75 eyes (47.47%) and 83 eyes (52.53%), respectively. The baseline characteristics of both groups are summarized in Table 1. There were no significant differences in sex (*p* = 0.568), age (*p* = 0.27), AL (*p* = 0.31), and SE (*p* = 0.961) between the two groups. The CFT of the VMT group was significantly higher than that of the ERM group (539.23 ± 183.49 vs. 472.59 ± 187.24 μm, *p* = 0.001). Moreover, the BCVA of the VMT group was significantly worse than that of the ERM group (0.94 ± 0.41 vs. 0.74 ± 0.46, *p* < 0.001).

**Table 1:** Comparation of demographics and ocular parameters between eyes with different vitreomacular interface abnormalities.

**Characteristics**	**Total**	**ERM group**	**VMT group**	***P***
Number, eyes/ patients	158/121	75/58	83/65	
Age, year	53.60 ± 10.63	52.48 ± 10.85	54.60 ± 10.23	0.27^#^
SE, diopter	−13.19 ± 4.41	−13.04 ± 5.54	−13.32 ± 4.27	0.96^†^
AL, mm	29.33 ± 1.59	29.50 ± 1.79	29.18 ± 1.37	0.31^†^
BCVA, logMAR	0.85 ± 0.45	0.74 ± 0.46	0.94 ± 0.41	<0.001^†^^*^
CFT, μm	507.60 ± 187.68	472.59 ± 187.24	539.23 ± 183.49	0.001^†^^*^

*ERM, epiretinal membrane; VMT, vitreomacular traction; SE, spherical equivalent; AL, axial length; BCVA, best-corrected visual acuity; logMAR, the logarithm of the minimum angle of resolution; CFT, central foveal thickness. P, comparison between the two groups*.

# *Independent sample t-tests*.

† *Mann–Whitney test*.

* *Significance at p ≤ 0.05*.

**Figure 2:**
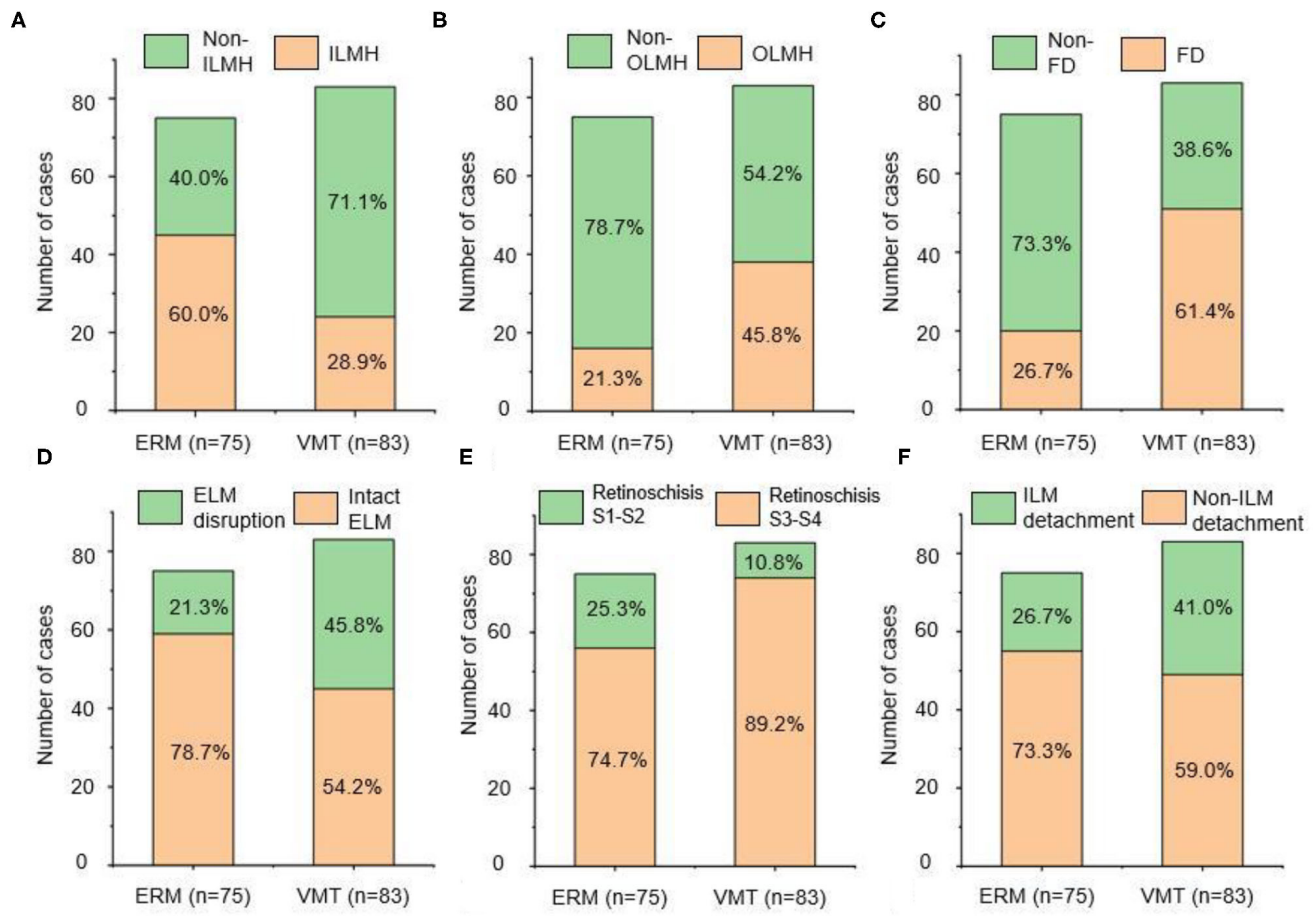
The prevalence of different anatomical configuration: **(A)** ILMH, **(B)** OLMH, **(C)** FD, **(D)** ELM integrity, **(E)** degree of retinoschisis, **(F)** ILM detachment. The left side of each figure presented the ERM group, and the right side of each figure presented the VMT group. ERM, epiretinal membrane; VMT, vitreomacular traction; ILMH, inner lamellar macular hole; OLMH, outer lamellar macular hole; FD, foveal detachment; ELM, external limiting membrane; ILM, inner limiting membrane.

The morphological characteristics of MF in the two groups were evaluated by OCT. As shown in Figure 2, the prevalence of foveal detachment was significantly higher in the VMT group compared with the ERM group (61.4 vs. 26.7%; *p* <0.001), so was the prevalence of OLMH (45.8 vs. 21.3%; *p* = 0.001). In contrast, a lower rate of ILMH (28.9 vs. 60.0%; *p* = 0.001) was detected in the eyes with VMT compared with those with ERM. The disruption of the ELM was more common in the eyes with VMT than in those with ERM (45.8 vs. 26.7%; *p* = 0.001). Similarly, more eyes with EZ disruption were detected in the VMT group than that in the ERM group (47.0 vs. 21.3%; *p* = 0.001). The distribution of outer retinoschisis grades was also different in the two groups. A higher rate of advanced retinoschisis (S3–S4) was found in the VMT group than that in the ERM group (89.2 VS. 74.7%; *p* = 0.017). The incidence of ILM detachment was higher in the VMT group compared with the ERM group, but the difference was not significant (*p* = 0.058). No significant difference was found in the presence of inner retinoschisis (*p* = 0.31), DSM (*p* = 1.00), and PS (*p* = 0.29) between the two groups (Table 2).

**Table 2:** Comparation of morphological characteristics between eyes with different vitreomacular interface abnormalities.

**Characteristics**	**Total (158 eyes)**	**ERM group (75 eyes)**	**VMT group (83 eyes)**	***P***
Inner LMH	69 (43.7%)	45 (60.0%)	24 (28.9%)	0.001^#^^*^
Outer LMH	54 (34.2%)	16 (21.3%)	38 (45.8%)	0.001^#^^*^
Foveal Detachment	71 (44.9%)	20 (26.7%)	51 (61.4%)	<0.001^#^^*^
ELM integrity	104 (65.8%)	59 (78.7%)	45 (54.2%)	0.001^#^^*^
EZ integrity	103 (65.2%)	59 (78.7%)	44 (53.0%)	0.001^#^^*^
Outer Retinoschisis Grading (S1-2/S3-4)	28/130	19/56	9/74	0.017^#^^*^
ILM Detachment	54 (34.2%)	20 (26.7%)	34 (41.0%)	0.058^#^
Inner Retinoschisis	93 (58.9%)	41 (54.7%)	52 (62.7%)	0.31^#^
Dome-shaped Macula	3 (1.9%)	1 (1.3%)	2 (2.4%)	1.00^†^
Posterior Staphyloma	13 (8.2%)	8 (10.7%)	5 (6.0%)	0.29^†^

*ERM, epiretinal membrane; VMT, vitreomacular traction; LMH, lamellar macular hole; ELM, external limiting membrane; EZ, ellipsoid zone; ILM, inner limiting membrane. P, comparison between the two groups*.

# *Chi-square test*.

† *Fisher exact probability test*.

* *Significance at P ≤ 0.05*.

To better evaluate the impact of VMIAs on the morphological characteristics of MF, subgroup comparison between the eyes with focal VMT (the area of attachment is 1500 μm or less) and broad VMT (the area of attachment is more than 1500 μm) was conducted. Among the 75 eyes with VMT, 32 eyes presented broad VMT and 51 eyes presented focal VMT. There was no significant difference in age, BCVA, AL, and SE between the focal VMT group and the broad VMT group. Similarly, no significant difference was found on the prevalence of foveal detachment, OLMH, and ILMH between the two groups (Supplementary Table S1).

### Data of Surgical Objects

A total of 61 eyes underwent vitrectomy. The mean follow-up time was 23.55 ± 19.92 (6–81) months. Both, the improvement of BCVA and the reduction of CFT were statistical after operation (logMAR 0.57 ± 0.40 vs. 0.92 ± 0.44, *p* = 0.007; CFT 118.74 ± 34.54 μm vs. 547.83 ± 154.50 μm, *p* = 0.007). Anatomical resolution was found in 51 eyes (83.6%) at final follow-up, 31 eyes of them achieved within 6 months. The mean time of resolution was 7.66 ± 4.55 months. After surgery, inner foveoschisis disappeared in all eyes (47 eyes, 100%) within one month, while the mean resolution time of outer foveoschisis and foveal detachment was 7.57 ± 4.49 months and 8.43 ± 3.57 months, respectively. After surgery, the final BCVA is statistically improved in 43 eyes, kept unchanged in 15 eyes, and deteriorated in three eyes (Table 3).

**Table 3:** Comparation of demographic and surgical data between eyes with different pattern of vitreomacular interface abnormalities.

**Characteristics**	**Total (61 eyes)**	**ERM group (27 eyes)**	**VMT group (34 eyes)**	***P***
Age, year	52.36 ± 10.25	52.52 ± 9.85	52.24 ± 10.69	0.916^#^
SE, diopter	−15.15 ± 4.56	−16.60 ± 4.82	−13.99 ± 5.12	0.302^†^
AL, mm	29.17 ± 1.65	29.42 ± 1.79	28.98 ± 1.52	0.305^#^
Follow-up period, month	23.55 ± 19.92	22.10 ± 14.69	24.70 ± 24.72	0.094^†^
Tamponade, air/C3F8	48/13	22/5	26/8	0.64^&^
Gauge, 23/25/27	6/39/16	3/18/6	3/21/10	0.86^‡^
BCVA, logMAR				
Pre-op	0.92 ± 0.44	0.88 ± 0.47	0.94 ± 0.41	0.258^†^
Post-op^a^	0.68 ± 0.33	0.71 ± 0.38	0.66 ± 0.53	0.279^†^
Post-op^b^	0.57 ± 0.40	0.65 ± 0.45	0.51 ± 0.36	0.326^†^
Improvement^b^	0.34 ± 0.42	0.24 ± 0.46	0.42 ± 0.36	0.149^†^
CFT, μm				
Pre-op	547.83 ± 1554.50	487.12 ± 89.14	596.04 ± 178.12	0.001^†^^*^
Post-op^a^	138.55 ± 58.45	128.64 ± 51.34	146.42 ± 73.52	0.788^#^
Post-op^b^	118.74 ± 34.54	119.69 ± 38.30^§1^	117.98 ± 31.86^§2^	0.852^#^
Reduction^b^	425.65 ± 156.54	367.21 ± 100.53	471.69 ± 177.64	0.007^†^^*^
Rate of vision improvement	60.7%	44.4%	73.5%	0.02^&^^*^
Rate of resolution within 6 months	50.8%	51.9%	50.0%	0.886^&^

*ERM, epiretinal membrane; VMT, vitreomacular traction; SE, spherical equivalent; AL, axial length; Pre-op, preoperative; post-op, postoperative; BCVA, best-corrected visual acuity; logMAR, the logarithm of the minimum angle of resolution; CFT, central foveal thickness*.

*P, comparison between the three groups*.

a *3 months after surgery;*

b *final follow-up*.

# *Independent sample t-tests;*

† *Mann–Whitney test*

& *Chi-square test*.

‡ *Fisher exact*.

§1 *Comparison between CFT at different timepoints in ERM group: F = 113.4; p < 0.001 by one-way ANOVA test*.

§2 *Comparison between CFT at different timepoints in VMT group: F = 107.2; p < 0.001 by one-way ANOVA test*.

* *Significance at p ≤ 0.05*.

**Figure 3:**
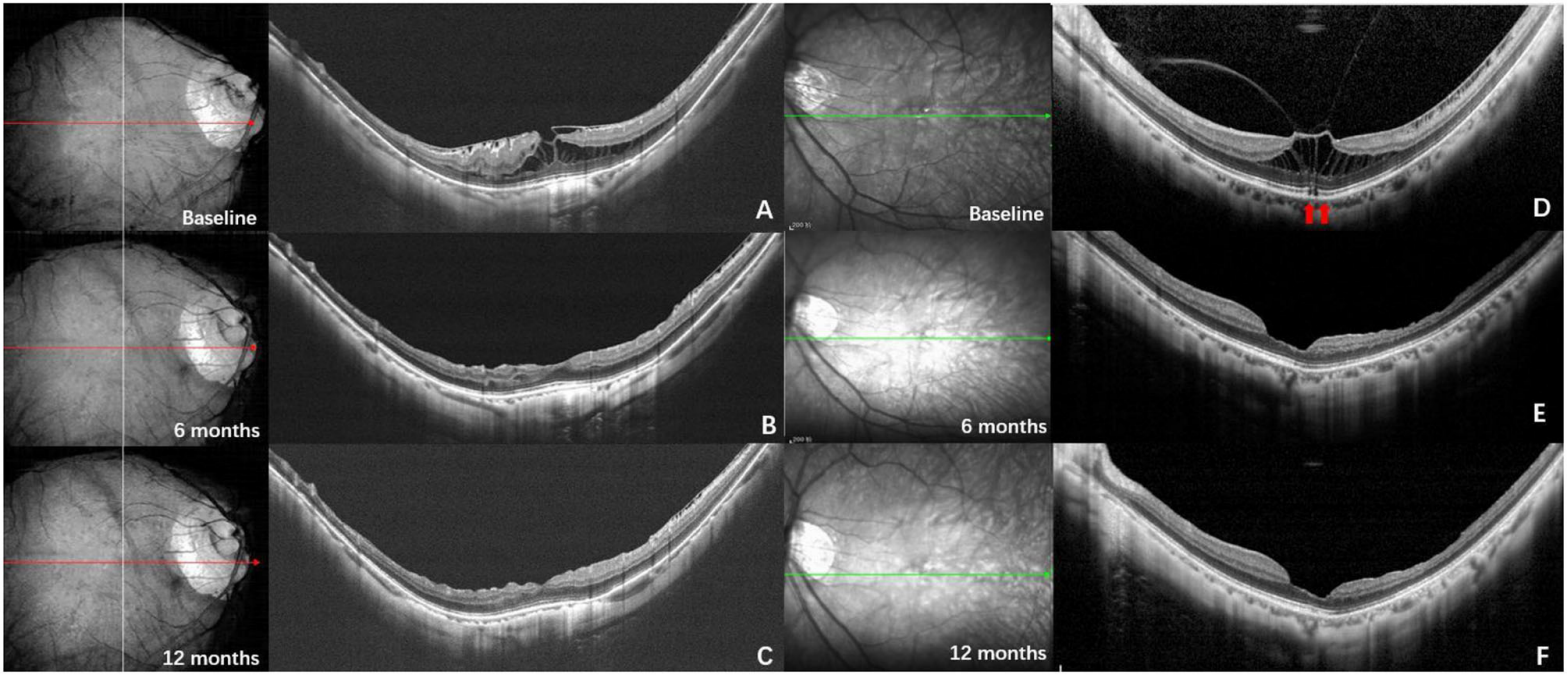
Typical surgical cases. (Left) OCT images of a 51-year-old man. **(A)** The OCT at baseline shows foveoschisis with the traction of epiretinal membrane (ERM). The axial length was 29.75 mm, and the baseline Logmar best corrected visual acuity (BCVA) was 0.3; **(B)** six months after vitrectomy, internal limiting membrane and ERM peeling and air tamponade, most of the retinoschisis resolved apart from the perifovea; **(C)** twelve months after surgery, the retinoschisis completely resolved, the Logmar BCVA was 0.05; (Right) OCT images of a 52-year-old woman. **(D)** The OCT at baseline shows foveoschisis with vitreoretinal traction (VMT). The ellipsoid zone of the outer retina was disrupted (red arrows). The axial length was 28.10 mm, and the baseline Logmar BCVA was 0.7; **(E)** six months after vitrectomy, internal limiting membrane peeling and air tamponade, the retinoschisis completely resolved, the ellipsoid zone became intact; **(F)** twelve months after surgery, the fundus remained stable, and the Logmar BCVA was 0.3.

Among the eyes that underwent surgical treatment, ERM and VMT were found in 27 (44.26%) and 34 (55.73%) cases, respectively. Typical surgical cases of both groups are displayed in Figure 3. There was no significant difference neither in the age, refractive error, and AL between the two groups (*p* = 0.916, *p* = 0.302, and *p* = 0.305), nor in the proportion of different tamponade (*p* = 0.64), gauge (*p* = 0.86), and the follow-up time (*p* = 0.094). No significant difference in the rate of anatomical resolution within 6 months postoperatively was found between the two groups (*p* = 0.886). The CFT reduction was significantly higher in the VMT group than that in ERM group (471.69 ± 177.64 μm vs. 367.21 ± 100.53 μm, *p* = 0.007). The eyes in VMT group had a higher rate of vision improvement (≥ 2 lines) than the eyes in the ERM group (*p* = 0.02) (Table 3).

Three eyes (1.90%) developed a full-thickness macular hole and one eye developed vitreous hemorrhage during the follow-up period. Their clinical data are presented in Table 4.

**Table 4:** Data of eyes developed postoperative macular hole.

**Case**	**1**	**2**	**3**
Sex	Male	Female	Female
Age, year	55	53	41
SE, diopter	−25	−18	−15.5
AL, mm	34.32	26.7	26.39
Follow-up period, month	8	12	16
VMIA	ERM	VMT	ERM
Foveal detachment	non-FD	FD	FD
Lamellar macular hole	ILMH	OLMH	OLMH
Tamponade	Air	Air	Air
Pre-op			
BCVA, logMAR	20/125(0.15)	20/125(0.15)	20/125(0.15)
CFT, μm	303.5	835.5	493
Post-op			
BCVA, logMAR	20/100(0.2)	20/167(0.12)	20/125(0.15)
CFT, μm	105.5	90	-
Time of MH formation	1 month	1 week	1 week
Second operation	Y	Y	N
Anatomical resolution	Y	Y	N(MH)

*VMIA, vitreomacular interface abnormality; ERM, epiretinal membrane; VMT, vitreomacular traction; FD, foveal detachment; ILMH, inner lamellar macular hole; OLMH, outer lamellar macular hole; SE, spherical equivalent; AL, axial length; Pre-op, preoperative; Post-op, postoperative; BCVA, best-corrected visual acuity; logMAR, the logarithm of the minimum angle of resolution; CFT, central foveal thickness; MH, macular hole*.

## Discussion

Our results showed that in eyes with MF, the pattern of the VMIAs and the damaged retinal layers were spatially related to each other. Retinal lesions in eyes with VMT predominantly affected the outer retinal area, whereas in eyes with ERM, retinal lesions predominantly affected the inner retinal area. To our best knowledge, this work firstly described the relationship between the subtypes of VMIAs and the morphological characteristics of MF, and connected it with the outcome of PPV. Tangential force in association with ERM may act as a causative factor for the inner retinal lesions in MF, whereas an inward-directed force resulting from VMT may act as a causative factor for the outer retinal lesions in MF. To better understand the force loading status of the retina under the condition of different VMIAs, we also set up a model to simulate the deformation and the forces of the posterior ocular wall.

The natural progression of MF is slow. It can remain stable for years without vision impairment in some cases. Gaucher et al. (10) reported 9 of 29 eyes with MF kept stable in visual acuity and CFT during the follow-up period of 12–59 months, they found that the presence of ERM and VMT may increase the risk of vision loss. From the perspective of mechanics, the tractional forces exerted on retina differs by the pattern of VMIAs. Epiretinal membrane contraction mainly exerts tangential force on retina, (11, 12) and partial posterior vitreous detachment usually results in oblique vitreomacular traction, which can be divided into a tangential and anterior–posterior traction (10, 13, 14). Our data shows that patients with VMT had a higher prevalence of foveal detachment and outer lamellar macular hole than those with ERM. It demonstrates that anterior–posterior traction force may be the leading cause of foveal detachment in MF, and if the force further exacerbated, outer lamellar macular hole may occur. Similarly, Tsai (15) demonstrated that the vertical tractional force results in greater structural changes in idiopathic MHs. PS and subsequent outward stretching play a role in the generation of MF (16–21). Shinohara et al. (21) found that patients with myopic retinoschisis have a significantly higher prevalence of PS (117/136, 86.0%) than those without (365/593, 61.6%), and they think posterior-directed force in association with staphylomas may contribute to the formation of myopic retinoschisis. This may be the reason for high prevalence of foveal detachment and outer lamellar macular hole in MF patients with VMT.

**Figure 4:**
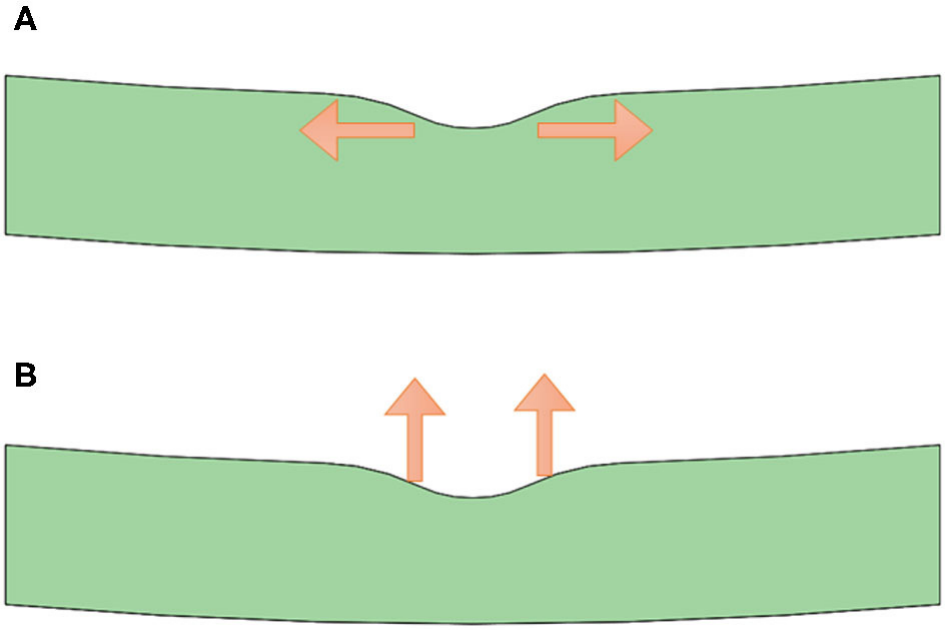
Structure model with forces of different directions applied. **(A)** Structure model with horizontal force applied (corresponding to the condition of epiretinal membrane); **(B)** Structure model with vertical force applied (corresponding to the condition of vitreoretinal macular traction).

To better understand the force-loading status of the retina under the condition of different VMIAs, we also set up a model to simulate the deformation and the forces of the posterior ocular wall. In our structure model, the eyeball radius is set as 1.2 mm. Note that the retinal thickness is set as 0.34 mm, whereas the central foveal has a thinner thickness, namely 0.27 mm. To simplify the simulation process, only the macula area was used for simulation. We used a commercial finite element modeling (FEM) software (Comsol Multiphysics 5.5) to simulate the stress distribution of the selected part of the retina. In detail, the structural mechanics module with a solid mechanics interface was chosen for our simulation, and the stationary state of the retina deformation under external load was investigated. We note that such external load in real world may come from the traction of the vitreous/epiretinal membrane and the traction direction can be diverse to induce different shape deformation of the retina. The structure model was first built in a 3D mechanical modeling software (Solidworks 2020), which was directly imported into the FEM software Comsol Multiphysics. The retina in the simulation model was considered as a linear elastic material, which is assumed to be isotropic. Therefore, only Young's modulus (0.2985 MPa) and Poisson ratio (0.4) (22, 23) of the retina were used as the input material parameters in the simulation model. In terms of the boundaries setting in the simulation model, fixed constraint was applied on the outer surface of the retina, whereas the boundary load was applied on the inner surface of the retina. In the first case (called “horizontal traction”), we applied one horizontal force (1 mN) toward the left side on the left-half part of the surface of the retina, whereas one horizontal force (1 mN) toward the right side was applied on the right-half part of the retina, as shown in Figure 4A. In the second case (denoted as “vertical traction”), these forces had an upward direction and the total force was summed as 2 mN, as shown in Figure 4B. As regards meshing of the model, we selected a tetrahedral shape as the mesh type, and the mesh size was set as extra fine. With the calculations controlled by the governing equation and boundaries and the following powerful postprocessing in Comsol Multiphysics, stress distribution of the retina under applied load can be obtained and clearly visualized.

**Figure 5:**
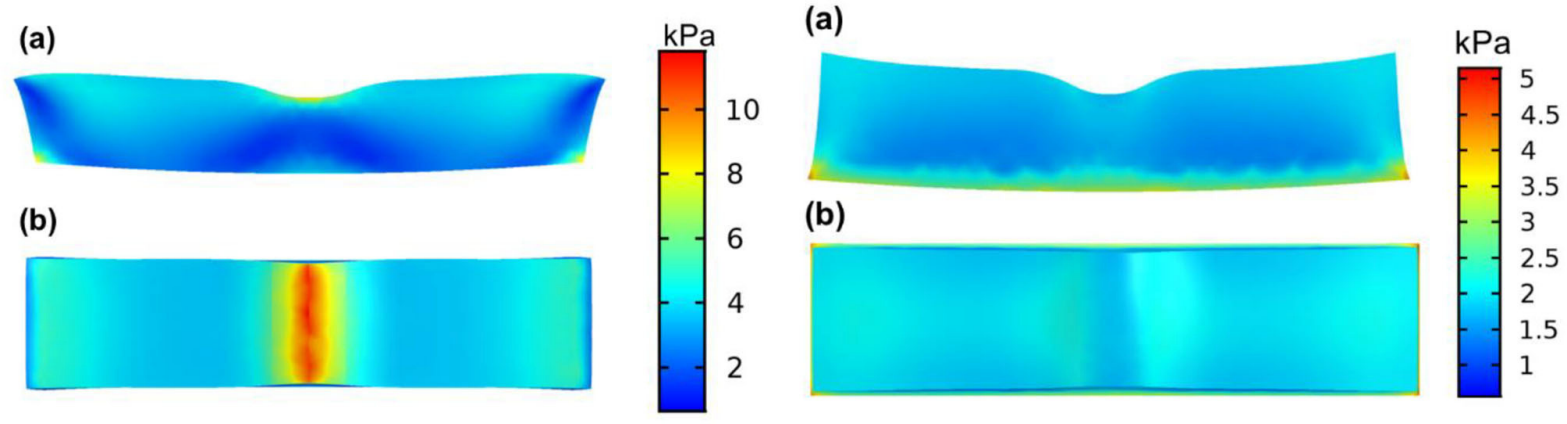
Stress distribution on the retina under forces of different directions applied. The left side shows the horizontal case model, simulating the epiretinal membrane condition. Under such loading condition, the maximum stress position located in the central vitreoretinal interface from the side view (Left **a**) and the top view (Left **b**), indicating the inner layer of the retina as the weakest point. The right side shows the vertical case model, simulating the vitreomacular traction condition. Under such a loading condition, a stress concentration at the outer retina was seen from the side view (Right **a**) and the top view (Right **b**), indicating the outer layer of the retina as the weakest point.

As shown in Figure 5, we note that stress concentration in the horizontal case is evident in the central position of the selected retina part, namely the fovea location. The underlying mechanism is that the foveal part has a thinner thickness (corresponding to a crosssection with smaller area), which is subject to higher stress under the same external load, compared with the inner retina with large-area cross sections. Furthermore, there exists a significant surface curvature variation in the fovea position, which tends to induce a stress concentration at the interface. Therefore, stress concentration occurs under such horizontal external load. Though the yield and crack processes are not considered in the current simulation model, it is reasonable to envision that inner retinal crack may appear under excessive load and more likely, fatigue cracks occur and ultimately lead to the breakage of the inner retina under continuous traction. The situation of the horizontal case agrees well with the ERM case, where ILMH usually occurs under traction by the ERM. Therefore, we would know that such case is likely subject to horizontal traction.

By applying an upward force on the retina, we learn that the stress distribution is quite different from the horizontal case, as shown in Figure 5. No obvious stress-concentrated positions are observed in the fovea part, whereas there exist evident distributions of large stress in the lower surface of the retina. Such stress distributions may indicate that the fovea is kept from stress-induced failure and cracks generation. In the vertical case, detachment of the retina is more likely to occur, considering the large-stress distribution in the whole outer surface of the retina. Similarly, the situation in the vertical case corresponds well with the condition of VMT, in which external layer damage of the retina is more common. Therefore, we expect a vertical traction by the vitreous in such situation. However, the current model was a possible mechanism behind the pattern of VMIA in MF. Further investigations on MF with VMIAs with long term follow-up are necessary to validate this theory.

Our study confirmed the curative effect of microincisional PPV with ILM peeling and gas tamponade for myopic foveoschsis. In our study, 83.6% (51/61) of the eyes achieved complete anatomical resolution at final follow-up, and postoperative visual acuity improved two lines or more in 60.7% (37/61) of the eyes. The resolution rate of MF reported in literatures ranged from 60 to 100% mainly due to the differences in surgical procedures and follow-up period (4, 24–31). Our data are in line with some studies on the subject. In the study of Wang et al., 33 eyes with MF were treated with PPV with ILM peeling and C3F8 tamponade, and after 6 months of follow-up, 76% (25/33) of eyes obtained complete resolution (29). Smiddy et al. reported that the visual acuity of 63% (10/16) of patients improved two lines or more after vitrectomy (30). Similarly, 64% (9/14) of patients in the study of Yamada et al. obtained a visual acuity improvement of no <2 lines (31).

This study is limited by its retrospective nature. Further prospective study is expected to explore the impact of VMIAs on the morphological features and surgical outcome of MF.

In conclusion, our results showed that in eyes with MF, the pattern of the VMIAs and the damaged retinal layers were spatially related to each other. Retinal lesions in eyes with VMT predominantly affected the outer retinal area, whereas in eyes with ERM, retinal lesions predominantly affected the inner retinal area. To our best knowledge, the present study firstly described the relationship between the subtypes of VMIAs and the morphological characteristics of MF, and connect it with the outcome of PPV. Tangential force in association with ERM may act as a causative factor for the inner retinal lesions in MF, whereas an inward-directed force resulting from VMT may act as causative factor for the outer retinal lesions in MF. Microincisional PPV with complete ILM peeling and gas tamponade is effective in the treatment of MF. By analyzing the distribution of the lesions and the subtypes of VMIAs in eyes with MF, and investigating the possible interplay between them by statistical methods and mathematical modeling, we believe our work will contribute to a better understanding of the mechanism of MF.

## Data Availability Statement

The original contributions presented in the study are included in the article/supplementary material, further inquiries can be directed to the corresponding author.

## Ethics Statement

The studies involving human participants were reviewed and approved by The Institutional Review Board of Shenzhen Eye Hospital (Shenzhen China). Written informed consent to participate in this study was provided by the participants' legal guardian/next of kin.

## Author Contributions

DF contributed to the study design and wrote the manuscript. LW contributed to the clinical data collection, data analyses, and manuscript polishing. LC contributed to manuscript preparation. JL assisted in clinical data collection. KL and XM assisted in data analyses. TX assisted in manuscript polishing. SZ contributed to the conception of the study, study design, and manuscript polishing. All authors read and approved the final manuscript.

## Funding

This work was supported by grants from the Sanming Project of Medicine in Shenzhen (SZSM202011015), the Natural Science Foundation of Guangdong Province (2021A1515011090), and the National Natural Science Foundation of China (81900877). These supporting organizations had no role in the design or conduct of the research.

## Conflict of Interest

The authors declare that the research was conducted in the absence of any commercial or financial relationships that could be construed as a potential conflict of interest.

## Publisher's Note

All claims expressed in this article are solely those of the authors and do not necessarily represent those of their affiliated organizations, or those of the publisher, the editors and the reviewers. Any product that may be evaluated in this article, or claim that may be made by its manufacturer, is not guaranteed or endorsed by the publisher.
