# Supplementary material for: Vitreomacular Interface Abnormalities in Myopic Foveoschisis: Correlation With Morphological Features and Outcome of Vitrectomy
Source: Front Med (Lausanne). 2022 Jan 5;8:796127. doi: 10.3389/fmed.2021.796127 (PMC8766811; doi:10.3389/fmed.2021.796127)
Supplement: Supplementary file 1 [file Table_1.docx]

**Supplementary Table S1 Comparison of Clinical Characteristics of Patients with Different VMT Subtypes**

| **Characteristics** | **Total** | **Broad VMT** | **Focal VMT** | **P** |
| --- | --- | --- | --- | --- |
| Number, eyes | 83 | 32 | 51 |  |
| Age, year | 54.60±10.23 | 59.38±8.56 | 51.60±9.30 | 0.88^#^ |
| SE, diopter | -13.32±4.27 | -11.92±4.10 | -14.20±3.32 | 0.44^†^ |
| AL, mm | 29.18±1.37 | 29.70±1.90 | 28.85±3.00 | 0.91^†^ |
| BCVA, logMAR | 0.94±0.41 | 0.89±0.34 | 0.97±0.61 | 0.60^†^ |
| CFT, μm | 539.23±183.49 | 440.23±123.36 | 601.35±167.35 | 0.10^†^ |
| Inner LMH | 24 (28.9%) | 10 (31.3%) | 14 (27. 5%) | 0.71^﹠^ |
| Outer LMH | 38 (45.8%) | 12 (37.5%) | 26 (51.0%) | 0.23^﹠^ |
| Foveal Detachment | 51 (61.4%) | 23 (71.9%) | 28 (54.9%) | 0.12^﹠^ |
| ELM integrity | 45 (54.2%) | 15 (46.9%) | 30 (58.8%) | 0.29^﹠^ |
| EZ integrity | 44 (53.0%) | 15 (46.9%) | 29 (56.9%) | 0.38^﹠^ |
| Outer Retinoschisis Grading  (S1-2/S3-4) | 9/74 | 3/29 | 6/45 | 1.00^‡^ |
| ILM Detachment | 34 (41.0%) | 11 (34.4%) | 23 (45.1%) | 0.33^﹠^ |
| Inner Retinoschisis | 52 (62.7%) | 22 (68.8%) | 27 (52.9%) | 0.15^﹠^ |

VMT, vitreomacular traction; SE, spherical equivalent; AL, axial length; BCVA, best-corrected visual acuity; logMAR, the logarithm of the minimum angle of resolution; CFT, central foveal thickness; LMH, lamellar macular hole; ELM, external limiting membrane; EZ, ellipsoid zone; ILM, inner limiting membrane;

P, comparison between the two groups;

^#^Independent sample t-tests; ^†^Mann–Whitney test; ^﹠^Chi-square test; ^‡^Fisher exact probability test; ^*^ Significance at P value ≤0.05
